# Addressing alcohol-involved sexual violence on college campuses: a collaborative system dynamics theory of change

**DOI:** 10.3389/fpubh.2025.1620598

**Published:** 2025-08-13

**Authors:** T. R. Moore, J. Alejandro, M. Dougherty, R. W. S. Coulter, J. G. Burke, E. Miller, R. Futcher, N. Sumetsky, C. F. Mair

**Affiliations:** ^1^Department of Nutrition Interventions, Communication, and Behavior Change, Friedman School of Nutrition Science and Policy, Tufts University, Boston, MA, United States; ^2^Department of Community Health, School of Arts and Sciences, Tufts University, Medford, MA, United States; ^3^Department of Behavioral and Community Health Sciences, School of Public Health, University of Pittsburgh, Pittsburgh, PA, United States; ^4^Center for Social Dynamics and Community Health, School of Public Health, University of Pittsburgh, Pittsburgh, PA, United States; ^5^Department of Pediatrics, University of Pittsburgh School of Medicine, Pittsburgh, PA, United States; ^6^Division of Adolescent and Young Adult Medicine, UPMC Children’s Hospital of Pittsburgh, Pittsburgh, PA, United States; ^7^Public Health Dynamics Laboratory, School of Public Health, University of Pittsburgh, Pittsburgh, PA, United States; ^8^Department of Epidemiology, School of Public Health, University of Pittsburgh, Pittsburgh, PA, United States

**Keywords:** alcohol, sexual violence, college, collaborative modeling, system dynamics, theory of change, policy

## Abstract

**Introduction:**

Alcohol-involved sexual violence on college campuses is a complex public health challenge shaped by interacting individual, interpersonal, social, and institutional factors. This paper presents a systems science–based theory of change for addressing alcohol-involved sexual violence, developed through collaborative modeling with campus collaborators as part of the CAMPUS (Collaborative Model-building Project to Understand Sexual Violence) study.

**Methods:**

This study presents a stock-and-flow diagram (SFD) theory of change developed by a research team through the synthesis of three causal loop diagrams co-produced by three cohorts of students and practitioners across five college campuses. The SFD formalizes key feedback structures shaping alcohol use, sexual violence, and campus responses.

**Results:**

The resulting SFD models the interplay of alcohol availability, drinking culture, peer norms, and consent communication. Key reinforcing loops highlight how alcohol use, consent, rape myths, and underreporting of sexual violence incidents mutually reinforce one another. Balancing loops underscore the potential of peer support and bystander interventions to interrupt these cycles. The model identifies multiple leverage points for systems-aligned interventions, including trauma-informed services and consent education.

**Conclusion:**

This practice-based and collaborative model provides a systems theory of change to guide future simulation modeling and intervention design.

## 1 Introduction

Alcohol-involved sexual violence is a persistent public health concern on college campuses, affecting a significant proportion of students, particularly women, LGBTQ+ individuals, and students of color (1–3). Although less frequently studied, male students are also affected by sexual victimization, with research suggesting that rates may be underestimated, due to underreporting and limited research attention (4). Despite decades of research and prevention efforts, rates of sexual violence remain high, with alcohol use and intoxication frequently cited as contributing factors in both exposure to and use of sexual violence (i.e, victimization and perpetration) (2, 5–7).

Traditional prevention strategies often emphasize individual- or interpersonal-level behavior change, such as risk reduction or awareness campaigns and bystander intervention trainings (8, 9). These approaches have largely shown limited success in addressing the broader system of interconnected social, cultural, and environmental drivers that sustain incidence of alcohol-involved sexual violence (10, 11). Recognizing this complexity and the need to consider social and structural interventions, public health researchers and practitioners are increasingly turning to systems science to better understand and intervene in the dynamic factors that contribute to campus sexual violence (10). Systems approaches allow for the examination of nonlinear feedback relationships, time delays, and unintended consequences, features often overlooked in linear intervention models (12).

Collaborative methods, such as community-based system dynamics, directly engage actors with different social backgrounds, expertise, and professional disciplines in identifying drivers of harm, co-developing causal models, and proposing context-specific solutions (13). These methods also build capacity within communities to understand and address complex problems beyond the scope of a single intervention. Parallel movements toward participatory engagement are evident in alcohol prevention efforts, where researchers have begun to recognize the importance of including students and other institutional collaborators in addressing the socio-emotional and environmental dynamics of alcohol use and its consequences (14, 15).

Emerging applications of systems science in sexual violence prevention are beginning to demonstrate the value of collaboratively developed models that reflect lived experiences, institutional dynamics, and social norms (10, 16). For example, the CDC’s STRIVE (Sexual Violence Prevention: Building Capacity and Promoting Equity) initiative encouraged grantees to use systems thinking tools (e.g., influence diagrams and root cause mapping) to engage community collaborators in identifying and addressing the structural drivers of sexual violence (17, 18). In a different context, Hovmand and colleagues applied community-based system dynamics to gender-based violence prevention in low-resource settings, facilitating group model building sessions with survivors and service providers to map feedback loops related to power, stigma, and help-seeking behavior (13). Systems principles have also begun to inform novel and promising bystander intervention efforts, such as the Green Dot program, where researchers recognized the importance of reinforcing social norms and peer dynamics in diffusing prevention messages across campus populations (19).

Despite these promising efforts, few public health studies have explicitly translated collaborative models into more formal system dynamics structures, like stock-and-flow diagrams (SFD), that can serve as actionable theories of change (ToC). A ToC is a structured framework that outlines how and why a desired change is expected to happen in a particular context. Developing a ToC using a SFD involves more than visualizing relationships among variables; it requires structuring the system in terms of key accumulations (stocks), the rates at which those accumulations change (flows), and the causal feedback mechanisms that drive system behavior over time. This level of formalization allows researchers and practitioners to go beyond descriptive models and instead build dynamic hypotheses that can be simulated, tested, and iteratively refined to inform strategic intervention design. In doing so, SFD-based theories of change provide a blueprint for understanding not just what is happening in a system, but how and why certain patterns persist or change in response to different inputs. This paper responds to the dearth of such systems dynamics theories of change in public health by presenting a model co-developed with students and campus practitioners across five campuses, focused on the dynamics of alcohol-involved sexual violence and how they might be disrupted through targeted, systems-informed interventions.

## 2 Methods

The SFD presented in this paper was developed through a collaborative process as part of the Collaborative Model-Building Project to Understand Sexual Violence (CAMPUS) study (10). This project engaged 39 collaborators (12 undergraduate students and 27 campus practitioners) from five college campuses in the Mid-Atlantic United States. Participants were purposively sampled to ensure diversity in age (students: 18–22 years, practitioners: 20–49 years), gender identity (including cisgender men and women, and genderqueer individuals), race/ethnicity (including White, Black/African American, Latine, Asian, American Indian/Alaska Native, and multiracial participants), and sexual orientation (including heterosexual, bisexual, pansexual, and other non-heterosexual identities). We also sought variation in student year, practitioner role (e.g., Title IX coordinators, health and prevention educators, student affairs staff), and institutional affiliation, which included a large community college, a small Catholic university, a small private university, a large private university, and a large public university.

The SFD builds upon a set of three causal loop diagrams (CLDs) co-produced through a series of four 2-h collaborative model building sessions held between April and November 2023, one cohort each for students and practitioners, with a third synthesis cohort. Sessions were facilitated by a core modeling team and guided by community-based system dynamics principles. In Session 1, participants brainstormed modifiable causes and consequences of alcohol-involved sexual violence across individual, interpersonal, campus, and societal levels. In Session 2, they used connection circles to map causal relationships among the most frequently cited variables. In Sessions 3 and 4, participants refined the CLDs, discussed feedback loops, and proposed and ranked interventions based on feasibility and impact. Between sessions, the modeling team translated visual outputs into digital CLDs using Kumu software and integrated literature to refine variable definitions and connections.

Importantly, this study did not replicate previous work (10); rather, we utilized the CLDs produced during the CAMPUS study, synthesizing themes to identify core system structures that informed the development of the SFD. While we reference Mair et al. (10) for detailed context, this manuscript expands upon that work by mapping dynamic feedback structures critical for simulation and strategy testing. The process of translating CLDs into an SFD involved identifying recurring patterns, prioritizing variables amenable to system-level intervention, and organizing them into stock, flow, and feedback structures that represent the accumulation and depletion of trust, exposure, and cultural norms over time.

SFD model development was carried out in Vensim (version 9.0.0) across multiple research meetings involving team members with expertise in systems science, public health, alcohol use, and sexual violence prevention (20). Discussions focused on clarifying causal relationships, refining variable language, and identifying feedback structures that best captured both collaborator narratives and evidence from existing research. A consensus process was used to finalize key reinforcing and balancing loops that form the backbone of the proposed theory of change.

Informed by both the literature and collaborator insights, an initial set of stocks was brainstormed to reflect accumulations in the system. Flows were iteratively added to represent how these stocks changed over time, to capture the influence of modifiable drivers such as alcohol availability, campus traditions involving alcohol, peer pressure, and coping through alcohol use. Feedback loops were developed to explain reinforcing dynamics (e.g., the normalization of drinking behavior) and balancing dynamics (e.g., improved consent communication reducing risk), and to identify leverage points for interventions and system-level change.

Throughout the modeling process, the research team systematically identified which components of the system could be feasibly quantified using available data or existing empirical studies (e.g., rates of alcohol consumption, prevalence of sexual violence, campus-level alcohol policies). We also explicitly noted elements for which quantification was currently infeasible due to limited data, ambiguous definitions in the literature, or lack of available scholarship in specific areas. These included concepts like harmful masculinity, support for survivors on campus, positive peer support, and campus traditions involving alcohol. These areas were clearly demarcated within the model structure to distinguish empirically grounded components from more conceptual or qualitative ones. The boundary-setting process helped define the scope of the current theory of change while identifying critical gaps in the literature where future research could expand the model’s empirical foundation.

To contextualize our ToC and demonstrate its utility in informing real-world programming, we applied the model to an environmental intervention implemented to reduce off-campus drinking and related harms: Safer California Universities (SaferCA) (21). This application highlighted the importance of multilevel interventions and illustrated how a ToC like the one we describe can help identify which feedback loops may be most proximal to influencing the outcome of interest: reductions in sexual violence incidents. By mapping elements of the SaferCA program onto our ToC, we showed how systemic patterns and leverage points can be understood and addressed through targeted, strategic actions.

## 3 Results

**Figure 1:**
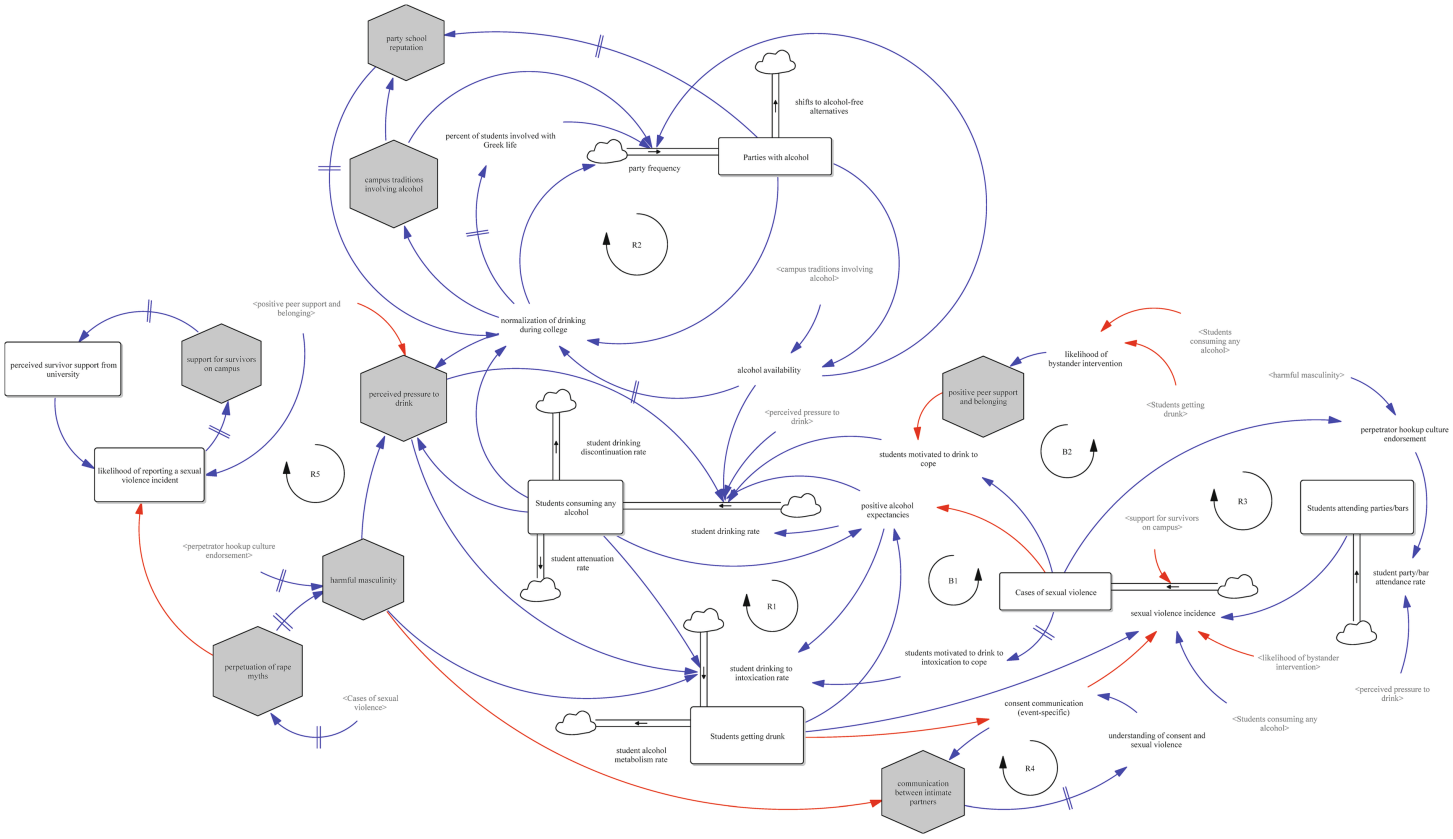
Full stock and flow diagram of alcohol-involved sexual violence on college campuses.

The resulting SFD represents a set of hypotheses of how alcohol consumption, sexual violence, peer norms, and campus systems interact to influence alcohol-involved sexual violence on college campuses. Seen in Figure 1, the model includes several key accumulations (stocks) such as students consuming any alcohol, parties with alcohol, students getting drunk, students attending parties/bars, and cases of sexual violence. These are shaped by flows such as student drinking rate, student drinking to intoxication rate, and student drinking discontinuation rate, and are influenced by interconnected reinforcing and balancing feedback loops. Together, these components reflect a complex, nonlinear system in which student behaviors, cultural norms, and institutional responses co-evolve over time.

To illustrate the function of the overall system, we present four views of the SFD, each highlighting key feedback loops that describe different subsystems contributing to alcohol-involved sexual violence. These include the reinforcing role of alcohol use, the cultural embedding of party norms, the interplay between consent communication and relationship dynamics, and the structural influences of rape myths and campus supports.

### 3.1 View 1: alcohol use and risk of sexual violence (reinforcing loop 1, balancing loop 1)

**Figure 2:**
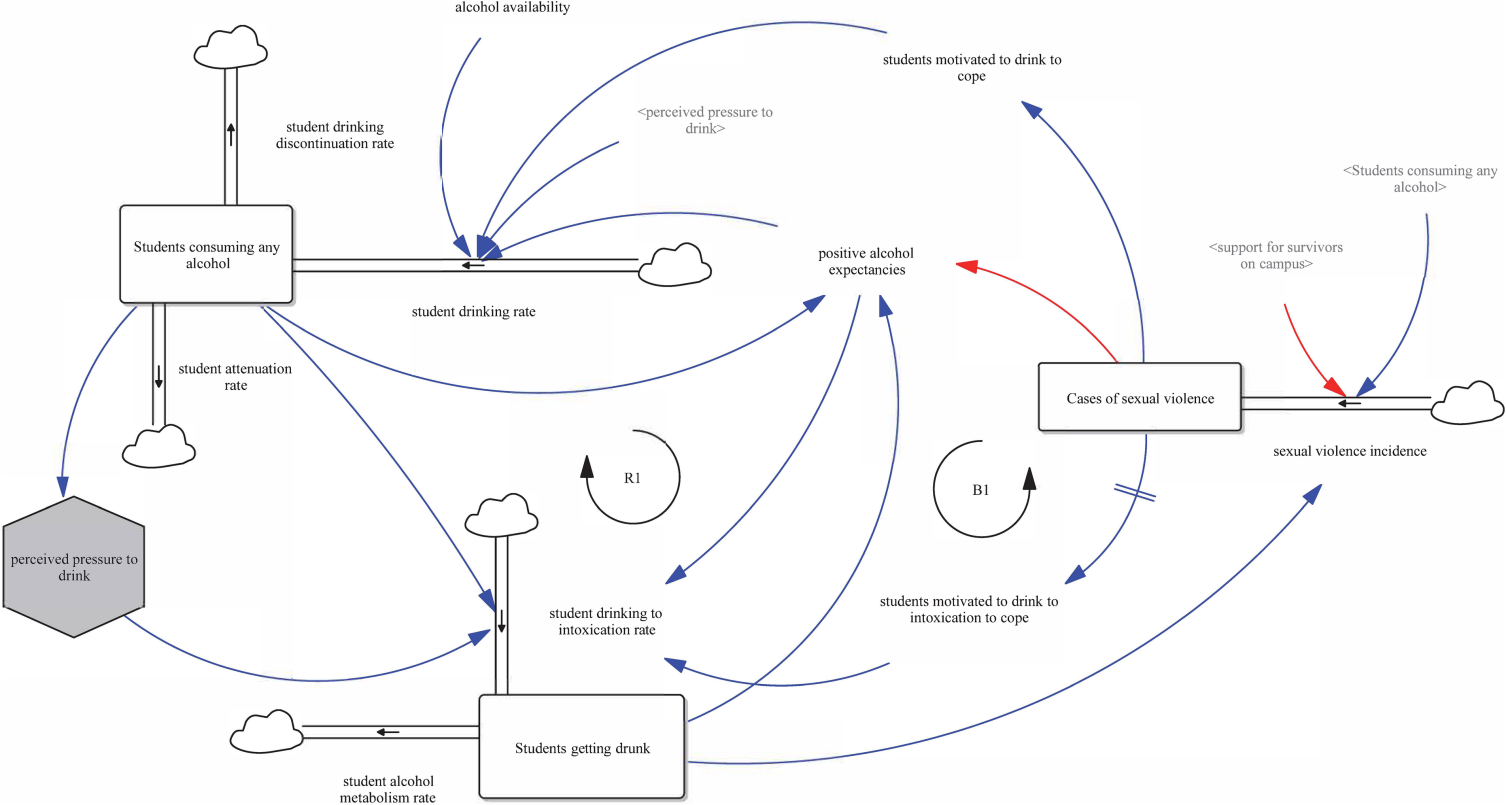
Alcohol consumption, sexual violence, and expectancies (Loops R1 and B1).

Figure 2 displays the feedback dynamics between alcohol consumption and sexual violence. Reinforcing loop R1 captures the cyclical relationship where increased alcohol use leads to greater intoxication, raising the risk of sexual violence. This is represented by an increase in the student drinking to intoxication rate (flow), which raises the stock of students getting drunk. This, in turn, increases the sexual violence incidence (flow), leading to a higher stock of cases of sexual violence. Affected students may respond by drinking to cope (22), reinforcing the overall student drinking rate (flow) and thus further increasing the stock of students consuming any alcohol.

Positive alcohol expectancies, defined in Supplementary Table 1 as beliefs in the perceived benefits of drinking, contribute to multiple causal pathways in the model. This variable increases perceived drinking benefits, which in turn elevates the student drinking rate. Through this mechanism, positive alcohol expectancies help drive reinforcing loops that link drinking behavior with risk of sexual violence.

The student alcohol metabolism rate functions as the outflow of the stock “students getting drunk,” representing the process by which students return to a sober state. This flow acts in opposition to the student drinking to intoxication rate and influences the duration that students remain in a state of intoxication, thereby moderating downstream risks.

Counteracting this, balancing loop B1 introduces a complementary mechanism. As cases of sexual violence increase, perceptions of risk associated with drinking may rise, reducing positive alcohol expectancies. This leads to a decrease in the student drinking rate, which dampens the upward spiral of R1. This illustrates how alcohol expectancies, as a moderating variable, may function both as a reinforcer within reinforcing loops and as a balancer when risk perceptions shift.

### 3.2 View 2: consent, communication, and relationship norms (reinforcing loop 4)

**Figure 3:**
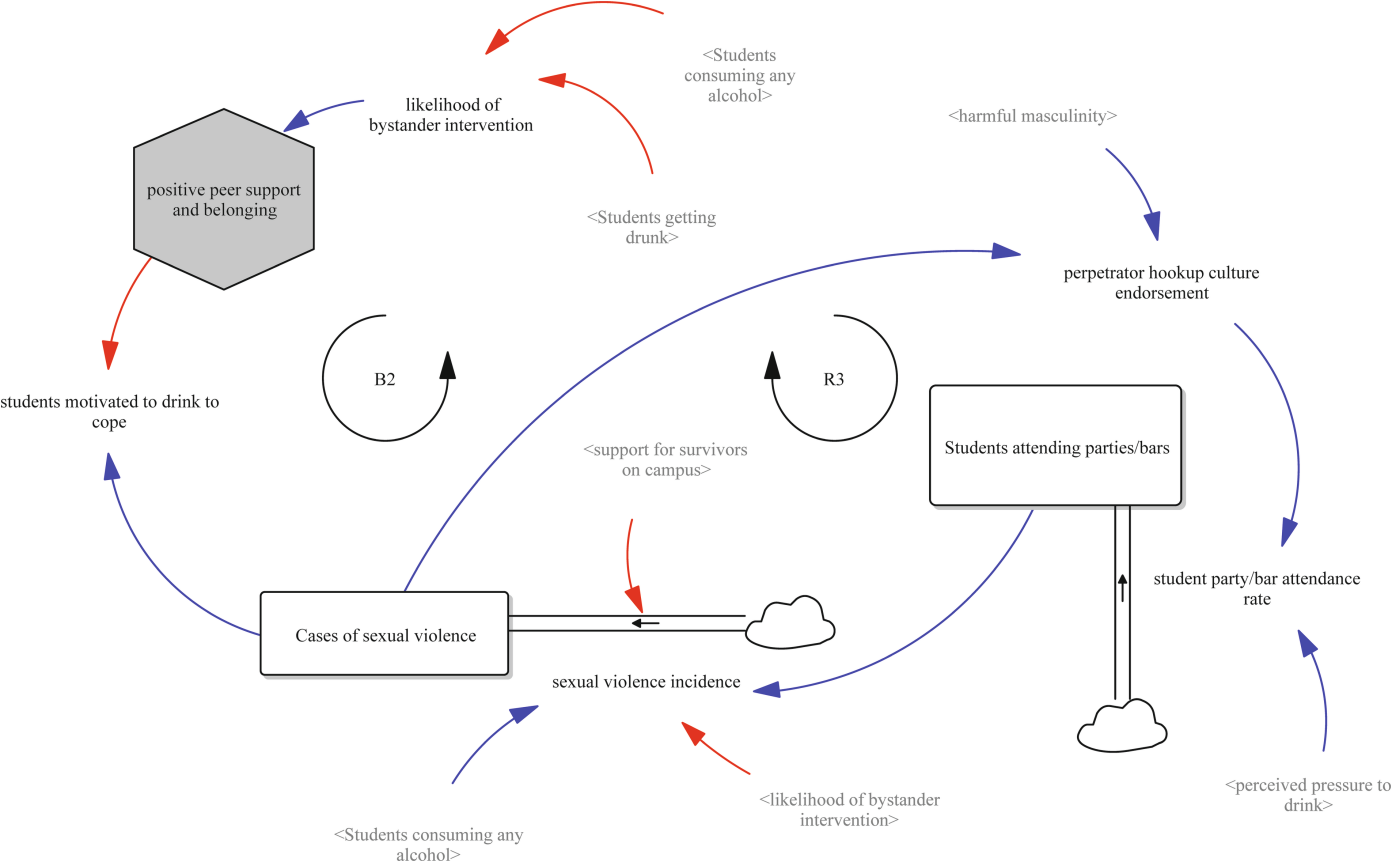
Intoxication, consent communication, and relationship norms (Loop R4).

Figure 3 illustrates the influence of drinking on interpersonal dynamics within intimate partnerships. Reinforcing loop R4 begins with the stock of students getting drunk, which directly reduces consent communication (event-specific). Reduced consent communication, in turn, leads to weaker long-term communication between intimate partners. Lower-quality long-term communication diminishes students’ understanding of consent and sexual violence over time. Understanding of consent and sexual violence influences event-specific consent communication, which we define as the occurrence of internal consent feelings and external consent communication prior to and during an intimate encounter. This conceptualization captures both clear and ambiguous expressions of consent, including instances where miscommunication occurs despite adequate understanding. As shown in the model, this variable interacts with students getting drunk and understanding of consent and sexual violence, influencing whether consent is clearly expressed and respected.

Harmful masculinity negatively influences long-term communication between intimate partners, weakening clarity, openness, and mutual respect within intimate relationships. This degraded communication reduces understanding of consent and sexual violence (auxiliary variable) over time, introducing a time delay between interpersonal dynamics and broader shifts in cultural awareness.

Additionally, the stock of students getting drunk negatively influences event-specific consent communication by impairing judgment and mutual clarity in high-risk scenarios, further compounding the effects of impaired communication pathways.

This loop illustrates how deeply embedded cultural and relational dynamics, particularly those influenced by gender norms and intoxication, can reinforce patterns of miscommunication and nonconsensual encounters. When left unaddressed, these dynamics persist through delayed feedback and weak corrective signals, emphasizing the need for multilevel interventions.

### 3.3 View 3: environmental risk and protective peer dynamics (B2, R3)

**Figure 4:**
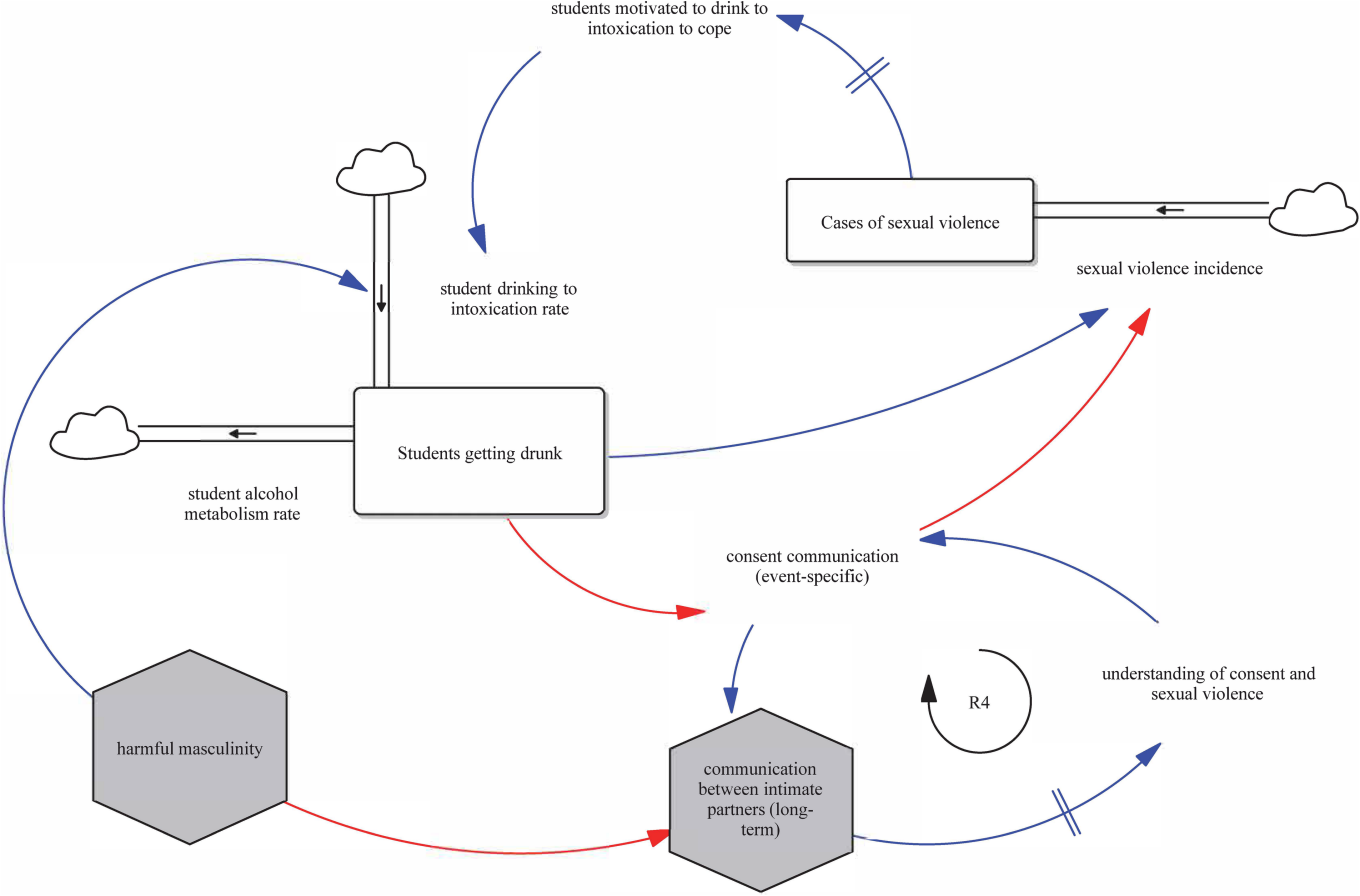
Risk environments, hookup culture, and protective peer influence (Loops R3 and B2).

Figure 4 focuses on the social and environmental settings where both risk and protection are shaped. Reinforcing loop R3 shows how perpetrator hookup culture endorsement increases the student party/bar attendance rate (flow), which raises the stock of students attending parties and bars. This elevates the sexual violence incidence (flow), increasing the stock of cases of sexual violence, which in turn prompts coping through alcohol use and reinforces the student drinking rate.

In contrast, balancing loop B2 offers a protective mechanism. Increases in the variable of positive peer support and belonging (i.e., the extent to which students feel loved, accepted, and socially connected to their peers) reduce perceived pressure to drink, which lowers the student drinking rate and subsequent student drinking to intoxication rate. This reduces both direct exposure to high-risk settings and downstream risks of sexual violence. Furthermore, strong peer networks increase the likelihood of bystander intervention, which further disrupts R3 and can help stabilize the system.

### 3.4 View 4: institutional and cultural drivers of campus drinking and sexual violence (reinforcing loop 2, reinforcing loop 5)

**Figure 5:**
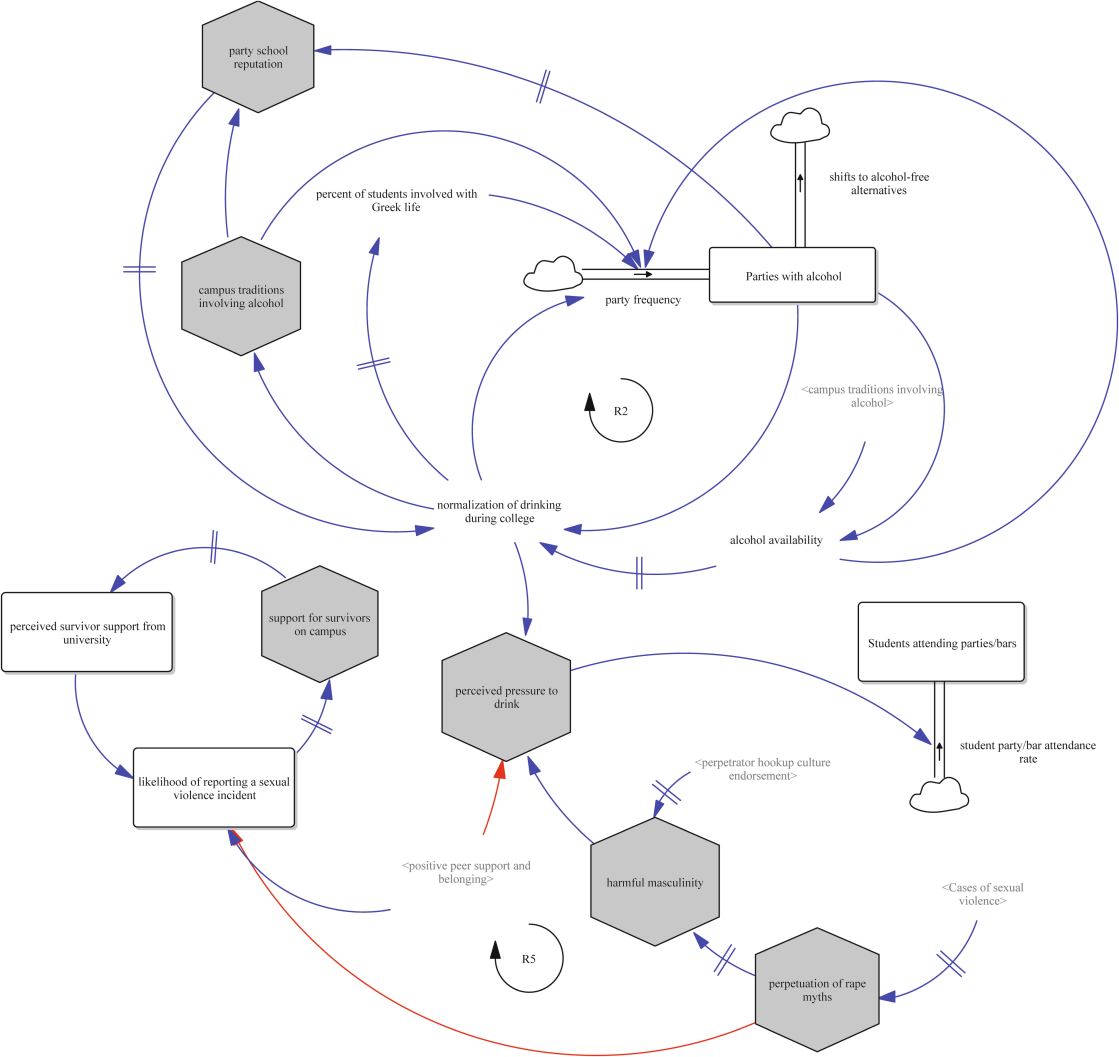
Institutional and cultural drivers of campus drinking and sexual violence (Loops R2 and R5).

Figure 5 highlights broader institutional and cultural dynamics. Reinforcing loop R2 shows how campus traditions involving alcohol and the percent of students involved in Greek life increase party frequency, raising the student party/bar attendance rate (flow) and the stock of students attending parties and bars. This further strengthens the campus reputation as a party school, increasing expectations for and normalizing drinking.

Reinforcing loop R5 illustrates how social and cultural dynamics can perpetuate sexual violence through interconnected feedback mechanisms. As the stock of sexual violence cases increases, it contributes—after a delay—to the perpetuation of rape myths. These myths, in turn, reinforce harmful expressions of masculinity, which are further intensified by the endorsement of perpetrator-centered hookup culture. Harmful masculinity increases the perceived pressure among students to drink in social settings, which elevates the rate of party and bar attendance. As more students attend these environments, the opportunity for alcohol-involved sexual violence grows, leading to an increase in the stock of sexual violence cases—thus completing the reinforcing loop. Notably, positive peer support and a strong sense of belonging can weaken this loop by reducing perceived pressure to drink. Several of the relationships in this loop—such as those between sexual violence and rape myth perpetuation, rape myths and harmful masculinity, and hookup culture endorsement and harmful masculinity—are characterized by time delays.

## 4 Discussion

This study offers a theory of change for addressing alcohol-involved sexual violence on college campuses using a systems science framework. The model draws on principles of collaborative model building and was co-developed with students and practitioners across five campuses. The model provides a structured, dynamic hypothesis of how alcohol use, social norms, and campus environments interact to shape the prevalence of sexual violence and where those patterns might be disrupted. A key strength of the model is its focus on modifiable risks, such as alcohol expectancies, perceived pressure to drink, or peer support, rather than fixed traits. This orientation enhances its utility for generating actionable, system-aligned recommendations that can be adapted across campus contexts.

Like other applications of systems thinking in public health, this model emphasizes the complex, nonlinear and interdependent nature of behavioral, social, and structural determinants as applied to alcohol-involved sexual violence. Feedback loops identified in this study reveal how alcohol consumption and sexual violence reinforce one another through mechanisms such as coping behaviors, normalized drinking culture, and campus environments that tacitly promote alcohol consumption. At the same time, the model identifies protective structures (e.g., positive peer support and alcohol-associated risk perception) that may be leveraged in future interventions for dampening these harmful cycles.

Consistent with previous collaborative model building applications in public health, such as Catalyzing Communities in the obesity prevention space (23, 24), this study illustrates how systems modeling can bridge qualitative insights with conceptual system structure. Further, the model enhances our ability to design interventions that align with the system’s behavior, rather than acting against it, by making visible the dynamics underpinning alcohol-involved sexual violence.

This model builds on prior systems-informed approaches in sexual violence prevention, such as those employed in the CDC’s STRIVE initiative (17). However, to our knowledge, it is among the first to convert collaborative systems mapping into a full SFD, offering a more formalized structure that can guide both simulation and strategic planning. The distinction between reinforcing and balancing loops allows for clearer identification of leverage points for intervention, as described below. These structures underscore how cultural, behavioral, and institutional variables operate not in isolation, but in feedback with one another.

Importantly, the SFD also distinguishes between empirically grounded elements and those that remain conceptual. This transparency helps clarify which relationships might be ready for simulation or quantitative evaluation, and where more research is needed to define variables, measure constructs, or validate hypothesized causal pathways. For instance, while variables like alcohol availability and reported incidents of sexual violence are measurable, constructs such as unhealthy relationship norms or party school reputation lack standardized measurement, limiting their immediate integration into formal simulation models. In many cases, the issue is not only a lack of available data, but also a lack of rigorous scientific inquiry into the mechanisms underlying these constructs, highlighting an urgent need for research that interrogates the cultural, interpersonal, and systemic drivers of sexual violence.

The collaborative development of this model also enhances its practical relevance. By grounding each loop in lived experiences and validating them through student and practitioner collaborator narratives, the resulting model reflects not only the system as it functions, but as it is experienced by those most affected.

### 4.1 Using feedback loops to identify potential interventions

Each feedback loop in the model reveals unique entry points for systems-aligned interventions. For example, reinforcing loop R5 illustrates how increases in sexual violence can, over time, contribute to the perpetuation of rape myths (25, 26). These myths reinforce harmful masculinity, particularly when shaped by perpetrator-centered hookup culture. This dynamic increases perceived pressure to drink, which leads to more frequent party and bar attendance and ultimately contributes to further incidents of alcohol-involved sexual violence. Interventions that promote positive peer support and a strong sense of belonging can help weaken this loop by reducing perceived pressure to drink (27, 28). Efforts to challenge harmful gender norms and reduce the social acceptance of rape myths, such as peer-led education, bystander training, and cultural campaigns, may also disrupt the reinforcing dynamics that sustain sexual violence on campus (28).

Reinforcing loop R1, which links alcohol consumption, intoxication, and sexual violence, suggests a need for multi-pronged approaches that reduce drinking to intoxication (29). Strategies such as social norms campaigns, alcohol-free late-night programming, and peer-led interventions have shown promise in reducing harmful drinking behaviors, particularly when paired with policies that limit alcohol availability at campus events (30–32). Integrating discussions of consent and sexual violence into alcohol education programs could also help reduce the normalization of intoxicated sex, a norm that reinforces this loop (33, 34).

Alcohol expectancies (i.e., individual beliefs about the effects of alcohol on social and sexual behavior) also play a central role in reinforcing cycles of risk. These expectancies can shape decisions to drink, influence perceptions of sexual behavior, and contribute to the normalization of intoxicated sex (35). As a modifiable factor, alcohol expectancies offer an important leverage point for campus interventions, particularly when targeted through norm-challenging campaigns or motivational interviewing strategies designed to reshape beliefs and reduce risky behaviors.

Reinforcing loop R4, which captures how intoxication erodes consent communication and fosters unhealthy relationship norms, points to the importance of comprehensive relationship and consent education. Programs like Bringing in the Bystander promote healthy communication skills, sexual ethics, and mutual respect, which can counteract the cumulative effects of miscommunication and harmful norms over time (36, 37). This loop also highlights opportunities to cultivate positive dynamics. Programs that support strong communication skills, mutual respect, and healthy expressions of masculinity can reinforce protective norms and foster relational environments where consent is actively practiced and understood.

In reinforcing loop R2, the connection between cultural drinking traditions and a school’s “party reputation” highlights the potential for environmental and policy interventions that shift campus identity over time and thus eventually reduce the “wetness” of a campus environment (38). Initiatives such as redesigning orientation to emphasize well-being and inclusion, partnering with student groups to create alternative traditions, and regulating high-risk events (e.g., limiting tailgates or pub crawls) can help dislodge alcohol from the center of campus social life (21, 39).

In contrast, balancing loop B2 emphasizes the protective role of peer support and bystander intervention. Interventions that foster positive peer influence, such as peer mentorship programs, campus-wide bystander training, and social belonging initiatives, can help reduce both drinking pressures and sexual violence risks (34, 40). These strategies may be designed specifically to address alcohol use or sexual violence, or they may more broadly aim to strengthen peer connection and support across campus. When they build authentic peer networks and are tailored to the needs and experiences of diverse student communities, these interventions are especially effective at reinforcing protective dynamics within the system (33, 41).

### 4.2 Applying the theory of change to safer California universities

The Safer California Universities study is a landmark randomized trial that tested a multicomponent environmental intervention implemented in 14 large public universities aimed at reducing student intoxication (and thus alcohol-related harms) in high-risk off-campus settings, including parties and bars (21). The program’s focus on enforcement strategies such as party patrols, DUI checkpoints, and minor decoy operations, along with efforts to increase the visibility of these actions, aligns with several elements of our ToC. In particular, it targets feedback mechanisms represented in reinforcing loop R2, where alcohol availability, cultural drinking norms, and student expectations interact to shape drinking behavior.

When viewed through the lens of the ToC, the SaferCA intervention demonstrates the potential for environmental-level strategies to influence system dynamics related to alcohol use. However, it also highlights opportunities to strengthen and expand intervention design by incorporating additional points of leverage identified in the model. While SaferCA focused primarily on enforcement and deterrence, it did not directly address cultural or relational drivers of drinking and sexual violence that are central to other feedback loops in the system. These include perceived pressure to drink, harmful masculinity, rape myth acceptance, and the influence of perpetrator-centered hookup culture.

The ToC offers a framework for identifying how interventions like SaferCA—which primarily focus on reducing alcohol-related harms but include secondary outcomes such as sexual violence and unprotected sex—could be expanded to more directly address sexual violence prevention. While SaferCA was not originally designed as a sexual violence intervention, its structure provides opportunities to build in sexual violence–specific elements. For example, integrating efforts that promote peer support and belonging could reduce perceived pressure to drink, as described in balancing loop B2. Additionally, enhancing the intervention with strategies that promote consent communication, challenge harmful gender norms, and reduce the social acceptance of rape myths could help interrupt reinforcing loops R4 and R5, which perpetuate cycles of alcohol-involved sexual violence.

### 4.3 Stock and flow diagrams as theories of change

The SFD presented here provides several key advantages to the CLDs often used by researchers as a theory of change to capture elements of system complexity and feedback (42). First, the SFD captures accumulation over time by modeling how stocks (such as the number of students getting drunk or cases of sexual violence) grow or decline in response to changing inflows and outflows. This distinction provides a more realistic depiction of how behaviors and risks accumulate and evolve over time, which is difficult to capture in a causal loop diagram CLD because CLDs do not represent stocks and flows. Second, the SFD helps transition from qualitative insight to dynamic simulation that can be used to test how interventions might affect system behavior under different conditions. Unlike CLDs, SFDs also differentiate between the current state of the system (stocks) and the drivers of change (flows and auxiliary variables), making causal logic and temporal dynamics more transparent.

Furthermore, SFDs help clarify the timing and sequence of potential intervention effects by illustrating how a change in one part of the system may propagate across others with delays or nonlinear intensity. The SFD’s explicit representation of time delays and accumulation processes allows researchers and practitioners to distinguish between proximal and distal predictors of sexual violence, a distinction well-established in behavioral science. Proximal drivers, such as event-specific consent communication or students consuming any alcohol, may offer immediate intervention targets, while more distal drivers (e.g., harmful masculinity, normalization of drinking during college) require sustained, systemic approaches. This capacity to differentiate interventions based on timing and impact sequence may add important nuance for implementation planning.

Finally, the SFD allows for greater precision in feedback loop structures, helping to distinguish between short-term versus delayed effects and making it easier to avoid misinterpretations of how quickly or strongly the system might respond to change. This matters for sexual violence interventions because it enables researchers and practitioners to estimate the likely impact, timing, and sustainability of interventions across interconnected factors (e.g., alcohol use, peer norms, and institutional response), thereby supporting more strategic and evidence-informed decisions about when, where, and how to intervene in order to achieve lasting change.

### 4.4 Limitations

The model reflects perspectives from five campuses, all in the same county in the Mid-Atlantic part of the United States, which may not capture the full diversity of campus environments, policies, or student demographics. Although the model emphasizes common dynamics across sites, future research could extend this work by exploring how local context moderates feedback structures (e.g., how historically Black colleges or commuter campuses may experience different cultural or institutional patterns). Additionally, while the SFD provides a strong conceptual foundation in the literature for both variables and direction of arrows (see Supplementary Table 1), quantifying it through dynamic simulation modeling would further strengthen its utility for forecasting intervention impacts and testing implementation strategies. Current limitations in both empirical data and scientific knowledge constrain the model’s development. In some cases, the necessary data do not exist or are not routinely collected. In other cases, the underlying mechanisms remain understudied or poorly understood, highlighting the need for more rigorous inquiry into the cultural, interpersonal, and institutional drivers of alcohol-involved sexual violence. Filling these gaps would significantly enhance the model’s accuracy, relevance, and practical value.

Although the collaborative process included diverse participants and perspectives, including that of the research team, many of the model’s dynamics could implicitly reflect dominant gender narratives (e.g., male perpetrator/female victim), which may limit its applicability to LGBTQ+ students or to male survivors. Future iterations of this model would benefit from explicitly examining how sex and gender shape pathways of risk and protection, including how masculinity norms, gender-based power imbalances, and experiences of harm differ across populations. Adapting the model to reflect these distinctions can uncover new leverage points and support the development of more inclusive prevention strategies.

Despite these limitations, this model provides a flexible, dynamic framework for understanding and addressing alcohol-involved sexual violence on college campuses. It can inform campus prevention planning, guide collaborator conversations, and support the co-design of interventions that consider not only immediate outcomes but downstream effects and unintended consequences. The SFD also aids in contextualizing social and structural interventions, which are often scarce both in the research literature and in campus prevention practices. Moreover, it demonstrates how systems science can contribute to a more holistic, context-sensitive, and action-oriented approach to addressing alcohol-involved sexual violence on college campuses, a pressing public health problem that has long seemed intractable.

## 5 Conclusion

This study presents a systems science–based ToC for alcohol-involved sexual violence on college campuses, grounded in collaborative modeling with students and staff. The ToC offers a structured, transparent, and flexible framework to identify reinforcing patterns of risk and opportunities for intervention by formalizing shared dynamics through a SFD. It advances the field by translating qualitative insights and collaborator knowledge into a system dynamics structure capable of informing policy and practice. As a next step, we will build an agent-based model to simulate how individual behaviors and interactions unfold within campus contexts over time. The agent-based model will allow us to test how different combinations of interventions influence system behavior across diverse student populations and campus environments, adding another layer of granularity and realism to our systems approach. Additionally, the agent-based model will be able to incorporate individual-level characteristics and explicitly model how individuals interact with other individuals and places across space and time; these interactions can elucidate emergent outcomes that are greater than the sum of their individual parts. Together, the SFD and agent-based model will provide a powerful toolkit for designing and adapting interventions that respond to the complexity of alcohol-involved sexual violence and support safer, more inclusive campus communities.

## Data Availability

The original contributions presented in the study are included in the article/Supplementary material, further inquiries can be directed to the corresponding author.
