# Supplementary material for: Addressing alcohol-involved sexual violence on college campuses: a collaborative system dynamics theory of change
Source: Front Public Health. 2025 Aug 13;13:1620598. doi: 10.3389/fpubh.2025.1620598 (PMC12380773; doi:10.3389/fpubh.2025.1620598)
Supplement: Supplementary file 1 [file Data_Sheet_1.docx]

**Supplementary Table 1. Stock and Flow Diagram Variables and Data Sources**

| **Variable** | **Definition** | **SFD representation** | **Data Source** |
| --- | --- | --- | --- |
| Alcohol availability | Amount of alcohol available for students on a given night or at a given location. | Auxiliary variable | Primary data – SaferCA(1) |
| Campus traditions involving alcohol | Specific formal or informal drinking events hosted on or close to campus (e.g., tailgates at sporting events, Greek rush, St. Patrick's Day, Halloween). | Auxiliary variable | Collaborator or expert opinion |
| Cases of sexual violence | Total events of sexual violence. | Stock | Primary data – GIFTSS/RAISE studies (2) |
| Communication between intimate partners | The presence of "good" communication around sexual preferences, including consent, between intimate partners. | Auxiliary variable | Collaborator or expert opinion |
| Consent communication (event-specific) | Occurrence of internal consent feelings and external consent communication prior to and during an intimate encounter. | Auxiliary variable | Primary data – GIFTSS/RAISE studies |
| Harmful masculinity | Detrimental societal and campus-level beliefs that perpetuate rigid, hetero-normative, violent, and controlling norms about what a “real man” is (3). | Auxiliary variable | Collaborator or expert opinion |
| Likelihood of bystander intervention | Likelihood of someone intervening if they witness the start of a sexual violence event. | Auxiliary variable | Primary data – GIFTSS/RAISE studies |
| Likelihood of reporting a sexual violence incident | Probability of reporting a sexual violence event to campus services by an involved party or another individual aware of the event. | Auxiliary variable | Published literature |
| Normalization of drinking during college | The campus-level perception that drinking is an acceptable and/or normal part of college. | Auxiliary variable | Primary data – Squad 2020 (4) |
| Parties with alcohol | Daily number of parties with alcohol. | Stock | Primary data – SaferCA |
| Party frequency | Parties with alcohol per day. | Flow | Primary data – SaferCA |
| Party school reputation | Reputation of a campus as a "party school" to people both on and off campus. | Auxiliary variable | Collaborator or expert opinion |
| Perceived pressure to drink | Extent to which students think others expect them to drink on a given night or at a given location. | Auxiliary variable | Collaborator or expert opinion |
| Perceived survivor support from university | Survivors' perceptions of institutional support following the disclosure of a sexual violence event. | Auxiliary variable | Published literature (5) |
| Percent of students involved with Greek life | Percent of students involved with Greek life on a campus. | Auxiliary variable | University administrative data |
| Perpetrator hookup culture endorsement | The extent to which sexual violence perpetrators endorse and participate in hookup culture. | Auxiliary variable | Published literature (6) |
| Perpetuation of rape myths | The number of students who endorse and sustain rape myths on a campus. | Auxiliary variable | Collaborator or expert opinion |
| Positive alcohol expectancies | The extent to which students believe their alcohol use will contribute to pleasant outcomes and be beneficial in some way. | Auxiliary variable | Published literature (7) |
| Positive peer support and belonging | The extent to which students feel loved, accepted, and socially connected to peers in relationships with open communication. | Auxiliary variable | Collaborator or expert opinion |
| Sexual violence incidence | Sexual violence events per day. | Flow | Primary data – GIFTSS/RAISE studies |
| Shifts to alcohol-free alternatives | Increase in the number of alcohol-free events held. | Flow | Published literature (8) |
| Student alcohol metabolism rate | Hourly rate at which a student’s BAC falls such that they are no longer intoxicated (e.g., Widmark Equation (9)). | Flow | Published literature |
| Student attenuation rate | Semesterly proportion of students graduating, disenrolling, or otherwise leaving campus. | Flow | University administrative data |
| Student drinking discontinuation rate | Daily proportion of students discontinuing alcohol use. | Flow | Primary data – Squad 2020 |
| Student drinking rate | Daily proportion of students drinking alcohol. | Flow | Primary data – Squad 2020 |
| Student drinking to intoxication rate | Daily proportion of students drinking to intoxication. | Flow | Primary data – Squad 2020 |
| Student party/bar attendance rate | Daily proportion of students attending parties or bars. | Flow | Primary data – Squad 2020 |
| Students attending parties/bars | Daily number of students attending parties or bars. | Stock | Primary data – Squad 2020 |
| Students consuming any alcohol | Daily number of students consuming alcohol. | Stock | Primary data – Squad 2020 |
| Students getting drunk | Daily number of students drinking to intoxication. | Stock | Primary data – Squad 2020 |
| Students motivated to drink to cope | Proportion of students motivated to drink to cope following an experienced sexual violence event. | Auxiliary variable | Primary data – Squad 2020 and published literature (10) |
| Students motivated to drink to intoxication to cope | Proportion of students motivated to drink to intoxication to cope following an experienced sexual violence event. | Auxiliary variable | Primary data – Squad 2020 and published literature |
| Support for survivors on campus | Extent to which a campus environment incorporates resources for sexual violence survivors, including university-sponsored resources provided by the university (e.g., trained trauma counselors) and a community of survivors (e.g., provide support to and advocate for their peers). | Auxiliary variable | Collaborator or expert opinion |
| Understanding of consent and sexual violence | The extent to which students understand what consent and sexual violence are. | Auxiliary variable | Primary data – GIFTSS/RAISE studies |

SaferCA, Safer California Universities study; GIFTSS/RAISE, Giving Information for Trauma Support and Safety/Reducing Alcohol Involved Sexual violence in higher Education studies

**References**

1. Saltz RF, Paschall MJ, McGaffigan RP, Nygaard PMO. Alcohol Risk Management in College Settings: The Safer California Universities Randomized Trial. Am J Prev Med. 2010 Dec 1;39(6):491–9.

2. Miller E, Jones KA, McCauley HL, Rofey DL, Clark DB, Talis JM, et al. Cluster Randomized Trial of a College Health Center Sexual Violence Intervention. Am J Prev Med. 2020 Jul 1;59(1):98–108.

3. Hill AL, Miller E, Switzer GE, Yu L, Heilman B, Levtov RG, et al. Harmful masculinities among younger men in three countries: Psychometric study of the Man Box Scale. Prev Med. 2020 Oct 1;139:106185.

4. Barnett NP, Light JM, Clark MA, Ott MQ, DiGuiseppi GT, Meisel MK. Dynamic social network analysis of a brief alcohol intervention trial in heavy-drinking college students shows spillover effects. Alcohol Clin Exp Res. 2024;48(2):375–88.

5. Graham AC, Mallinson RK, Krall JR, Annan SL. Sexual Assault Survivors’ Perceived Helpfulness of University-Affiliated Resources. Violence Women. 2021 Aug 1;27(10):1758–73.

6. Spencer CM, Rivas-Koehl M, Astle S, Toews ML, Anders KM, McAllister P. Risk Markers for Male Perpetration of Sexual Assault on College Campuses: A Meta-Analysis. Trauma Violence Abuse. 2023 Oct 1;24(4):2486–97.

7. Stamates AL, Preonas PD, Linden-Carmichael AN, Junkin E, Roberts R, Lau-Barraco C. Daily positive alcohol expectancies and alcohol use outcomes among college students. Addict Res Theory. 2024 May 3;32(3):219–24.

8. Wei J, Barnett NP, Clark M. Attendance at alcohol-free and alcohol-service parties and alcohol consumption among college students. Addict Behav. 2010 Jun 1;35(6):572–9.

9. Watson PE, Watson ID, Batt RD. Prediction of blood alcohol concentrations in human subjects. Updating the Widmark Equation. J Stud Alcohol. 1981 Jul;42(7):547–56.

10. Nelson JD, Fischer S. Recent Sexual Assault Predicting Changes in Coping Motives for Alcohol Use in First-Year College Women. Violence Vict. 2021 Jun 8;36(3):424–35.
